# Affiliative Behaviour and Conflictual Communication during Brief Family Therapy of Patients with Anorexia Nervosa

**DOI:** 10.1371/journal.pone.0070389

**Published:** 2013-08-01

**Authors:** Karyn Doba, Laurent Pezard, Guillaume Berna, Jean Vignau, Jean-Louis Nandrino

**Affiliations:** 1 Research Unit on Cognitive and Affective Sciences, Department of Psychology, University of North of France, Villeneuve d'Ascq, France; 2 Medical and Psychological Clinic, Foundation Health Students from France, Villeneuve d'Ascq, France; 3 INS, UMR 1106, Aix-Marseille University - Institut National de la Santé et de la Recherche Médicale, Marseille, France; 4 Addictology Department, Centre Hospitalier Universitaire of Lille, Lille, France; Catholic University of Sacred Heart of Rome, Italy

## Abstract

**Objective:**

Although patients with anorexia nervosa (AN) present positive responses to family therapy, the key features of therapeutic changes still require identification. This study explores the role of conflictual communication and affiliative nonverbal behaviour in therapeutic change in brief strategic family therapy (BSFT) for AN patients.

**Methods:**

Ten female AN patients and their parents were included in the sample and took part in a 6-month follow-up of BSFT. The durations of conflictual communication and of affiliative nonverbal behaviour estimated by eye contact were compared between the first and the last sessions of family-based treatment using nonparametric statistical tests.

**Results:**

An increase of the Body Mass Index associated with an increase in the conflictual communication expressed during BSFT sessions were observed. Moreover, affiliative nonverbal behaviour expressed by the father and the patient decrease, after a BSFT follow-up, in conflictual situations only. By contrast, no significant difference was observed in affiliative nonverbal behaviour expressed by the mother.

**Conclusion:**

The present study demonstrates that the impact of the BSFT differs between members of a family: the AN patient and the father have established a new form of emotional functioning with a decrease in emotional involvement. The study of the combination between verbal and nonverbal communication can represent an important step in the understanding of the mechanisms of therapeutic change.

## Introduction

Several follow-up studies of patients with anorexia nervosa (AN) have shown positive responses to family therapy [1]–[4]. Studies of AN patients have described the impact of family interventions that directly mobilize the family resources in tackling anorexic behaviours [5], [6]. Yet, the key features of these therapeutic changes still require identification and characterisation. Specifically, the critical variables involved in the mechanisms leading to the improvement of nutritional and psychological states of AN patients still need to be defined [5], [7]. As conflictual communication and nonverbal behaviour in families of patients with psychiatric disorders have been considered as fundamental components of interaction processes [8]–[10], they constitute important variables for revealing key features of therapeutic changes in family therapy. The monitoring of the evolution of these two components of interaction processes should thus enable one to identify and characterise therapeutic changes in familial interactions that appear during family therapy of AN patients.

From a general point of view, conflictual communication can be considered as interactions between persons that express opposing interests, views or opinions [11]. In the family therapy context, conflictual communication is defined in terms of disagreement, overt opposition, verbal aggression, criticism and arguments relating to the illness of patient and family relationships [12], [13]. Family systems theory has suggested that conflictual communication may help family members to renegotiate their roles and relationships and thus play an important part in the transition from hierarchical relationships at the outset of adolescence to egalitarian relationships at the outset of adulthood [14], [15]. The study of conflictual communication has been associated with those of expressed emotions in families of AN patients and have been used to infer the mechanisms of therapeutic changes during family therapy [6], [16]. The expressed emotions include critical comments, emotional over-involvement and hostility. These expressions are used to assess relatives emotional involvement towards a symptomatic family member and are evaluated by standard semistructured interviews [17], [18]. Although, it has been shown that parents express low levels of criticism and hostility towards AN patients [6], a long-term follow-up has demonstrated that decrease in both emotional over-involvement and hostile parental attitudes towards the illness are important factors for change in family interactions and for improvement of eating symptoms during family therapy [6], [19]. The effects of family therapy on family mechanisms have also been characterised using problem solving tasks and both self-reported and observer ratings of family conflict in outpatient treatment [20]. After a one-year follow-up of family therapy, a reduction in conflict specifically related to eating [20] and a change from covert to overt expression confrontation in general family relationships [16] have been reported in families of AN patients. Moreover, the increase in overt confrontation in the conflictual communication during family therapy predicted the improvement in the nutritional and psychological states of AN patients. These findings clearly suggest that conflictual communication is a significant and meaningful clinical measure of therapeutic change in family interactions during family therapy.

Conflictual communication produces emotional responses in participants that are reflected in affiliative nonverbal behaviour [21]–[23] which corresponds to an involvement characterised by high emotional engagement with another person [24], [25]. These affiliative nonverbal behavior are usually measured by eye contact [26], [27]. Indeed, an association between poor ability to inhibit eye contact and failure to differentiate self from other within a close relationship at the level of perceptions, opinions and emotions has been observed [28]. Recent studies showed that the ability to inhibit the gaze, characterized by decreased eye contact, predicts a decrease in emotional involvement in interpersonal or family relationships [27]–[29] and adaptive social functioning in adulthood [30].

From a therapeutic perspective, these studies suggest that family therapy may improve the conflictual communication skills of families by promoting a decrease in emotional involvement during these situations. Therefore, the affiliative behaviour revealed by eye contact could be important variable to monitor during therapeutic interventions in order to assess emotional involvement during conflictual communication. The present study reports the evolution of both conflictual communication and affiliative nonverbal behaviour in families with AN patients during brief strategic family therapy (BSFT) [31], [32]. The affiliative nonverbal behaviours expressed during conflictual and non-conflictual communication were considered to be signs of emotional involvement and were recorded during sessions of family therapy. We hypothesised that conflictual communication in family relationships increases at the end of BSFT and that affiliative nonverbal behaviour decreases during the conflictual communication. We also investigated whether the evolution of conflictual communication expressed during BSFT sessions were correlated with the clinical improvements evaluated with the Body Mass Index (BMI) and the scores to Morgan and Russell (MR) scales [33].

## Methods

This study was approved by an independent ethics committee (University of Lille, France) and adhered to the tenets of the Declaration of Helsinki.

### Procedure

The selection and inclusion of the families was conducted at the Addictology Department of the University Hospital (CHRU) of Lille (France). Each participant received a study information sheet and provided her written, informed consent to participation. The patients and their parents were informed about this study by a psychiatrist at admission to the Department of Addictology in our inpatient unit. The parents were all aware of their child's difficulties prior to their participation in the study. Prior to inclusion in the study, all patients were hospitalized in our inpatient unit for life-threatening physical and/or mental states. After, written consent was given by the patients and their parents, the therapists met the parents and patient in order to define the therapeutic objectives of the brief strategic family therapy (BSFT).

The first session of BSFT occurred in post-hospitalization where the patients received an outpatient treatment at Department of Addictology. In outpatient treatment, all patients received the same supportive medical care by psychiatrists and physicians.

### Participants

Ten families were included in the sample and participated in the study. They included the father (mean age: 49.4 Y.O., SD: 5.48), the mother (mean age: 49.3 Y.O., SD: 6.31) and the AN patient (mean age: 20 Y.O., SD: 2.27). The patients resided at home with both parents. Socioeconomic status of families was divided into three levels: 50% from social classes I and II, 40% from social classes III and V and 10% from social classes from VI and VIII [34], [35].

The female outpatients were selected according to the DSM-IV criteria for anorexia nervosa restrictive type, i.e., without the binge-eating and bulimics [36]. Diagnostic assignment was determined by the consensual judgment of two professionals (a psychiatrist and a clinical psychologist). Exclusion criteria were as follows: neurological disorders, intellectual deficits and a recent history of drugs or alcohol abuse. Axis II of the DSM-IV related to personality disorders was not assessed. The Body Mass Index (BMI) of the patients (mean: 13.9, SD: 1.07) and the duration of their eating disorders (mean: 2.9 years, SD: 1.52) were evaluated. All patients received antidepressant drugs (serotoninergic agonists).

### Treatment

As in the structural family therapy tradition [37], [38], the goal of BSFT is to alter specific patterns of dysfunctional interaction that maintain symptoms [31], [32], [39]. Investigations into the change processes that occur over the course of structural family therapy are notably absent [7], [40]. In families of AN patients, restructuring typically involves several overlapping processes:

1. Enmeshment is modified by increasing the interpersonal distance between over-involved individuals and by creating a stronger boundary between the parental subsystem and the sibling subsystem;
2. Detouring is reduced by helping the marital subsystem reduce focus on the problems of the anorexic patient;
3. Transactional rigidity is reduced by encouraging greater flexibility in modes of communications;
4. Restructuring also involves enhancing the family's ability to engage in and resolve conflict;
5. Fostering positive parenting and parental skills.

Family therapy was conducted by a therapist over a period ranging from five to six months and a frequency of one session per month (mean: 5.9, SD: 1.1). Each session lasted one hour and was videotaped. The treatment was terminated on the basis of the mutual decision of family members and the therapist. The two therapists (psychologists) involved in the study had more than six years of experience in the family therapy of AN patients.

### Assessment

#### Individual assessment

Semi-structured interviews allowed to obtain details of the history and clinical manifestations of the illness as well as the psychosocial functioning of the patient [33], [41]. The Morgan and Russell scales [33] (MR scales) were used to rate the patients' nutritional status (MRnut), menstrual functioning (MRmens), mental state (MRment), psycho-sexual adjustment (MRpsex) and psychosocial adjustment (MRpsoc). The average of these five scores was used as an average outcome score (MRave) rating from 0 to 12 with 12 indicating normal functioning. We reported weight and changes in weight as Body Mass Index (BMI in kg/m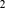).

At the end of family treatment, each patient's overall outcome was categorised using the following classification [6], [33]:

**Good outcome:** Patients in whom menstruation had returned with no evidence of bulimic pathology and whose weight was within 15% of the average body weight (20 kg/m^2^≤BMI≤23 kg/m^2^).

**Intermediate outcome:** Patients who had reached a normal weight but without the return of menstruation or who reported bulimic symptoms.

**Poor outcome:** Patients whose BMI was below 18 kg/m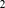.

Individual assessments were conducted by a clinical psychologist independent of the BSFT. Individual assessments were performed a week before BSFT and at the end of BSFT.

#### Assessments of family interactions

Family interactions were assessed off-line using videotaped recordings of two sessions: an initial session (Si ) at the beginning of BSFT and a final session (Sf ) at the end of BSFT. Verbal communication between family members were rated for conflictual/non-conflictual content [20], [27] as described in table 1. Overt expressions of confrontation were rated as conflictual communication, whereas agreement and positive appreciations were rated as non-conflictual communication. As it is very difficult to assess the value of a silence without ambiguity, we have considered that silence corresponds to an absence of overt expression of confrontation in communication and were also rated as non-conflictual communication [8].

**Table 1:** Operational definitions of the encoding categories for conflictual and non-conflictual verbal communication between family members.

Encoding categories	Practical description
Conflictual communications	Opposition to someone else or a disagreement
	Confrontation with another person
	Sarcasm, blame, disapproval, critical comments
Non-conflictual communications	Agreement with someone else
	Avoiding strategies
	Expression of solidarity and positive appreciation

Avoiding strategies include: change topic, stop communicating to get away from the issue.

Affiliative behaviours of the family members were assessed using the eye contact measure [27]. Eye contact toward another family member was considered as *intra-family* affiliation and eye contact toward the therapist was considered as *extra-family* affiliation. The absence of the eye contact with another family member or therapist and the orientation of the gaze to one's feet, the wall or the floor were classified as *non-affiliative behaviour*.

The encoding procedure was tested for inter-rater agreement. The inter-rater agreement was assessed on the two possible encoding categories of verbal interaction behaviour and on the four possible encoding categories of nonverbal behaviour. Seven family therapy sessions were separately encoded by two clinical psychologists independent of the BSFT. The Kappa-coefficients of inter-rater agreement of the verbal interaction behaviour (median score: 0.64) and the nonverbal behaviour (median score: 0.59) were considered satisfactory [42].

For each family and each session, our initial measurements correspond to the duration (in sec.) of eye contact from a family member to four possible targets: the two other family members (intra-family affiliation), the therapist (extra-family affiliation) and other targets (non-affiliative behaviour). These measure were obtained for both conflict and non-conflict communication. We denote 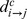 and 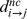 the duration of eye contact from participant 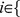
Patient, Father, Mother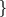 to 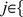
Patient, Father, Mother, Therapist, Other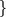 and 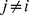 in conflict situations and non-conflict situations respectively. For the sake of notation simplicity, we will drop the 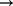 sign in the subscript from now on. From these measurement, we derived several indices to assess the evolution of conflictual communication and affiliative nonverbal behaviour between Si and Sf.

Due to the encoding strategy, the duration 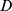 (in sec.) of the session is separated into conflictual and non-conflictual duration:
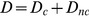
(1)and 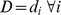. The duration 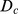 (in sec.) of conflict is:
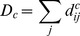
(2)and 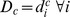. The duration 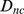 (in sec.) of non-conflict is:
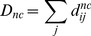
(3)and 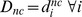.

The evolution of conflictual communication between Si and Sf in the families of AN patients was characterised using the fraction of the session duration with conflictual communication denoted 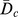 and defined as follows:
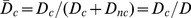
(4)


Since the fraction of non-conflict communication (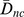) adds to one with 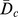, these indices are redundant and 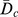 is only used here.

The evolution of affiliative behaviour was assessed independently for each family member and for conflictual and non-conflictual communication. For each family member 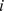, we computed the fraction of the condition duration devoted to one specific eye contact as:
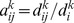
(5)where 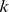 is either 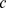 (conflictual) or 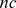 (non-conflictual) and 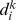 has the same meaning as in Equations (2) and (3). We thus have: 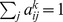. For each family, we thus ended with four matrices i.e. for conflictual and non-conflictual situations at Si and Sf of the following form:
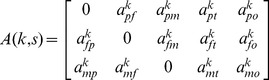
(6)where 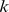 is either 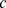 or 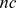, 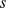 is either Si or Sf and 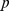, 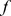, 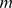, 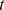 and 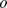 stand respectively for patient, father, mother, therapist and other.

### Statistical Procedures

The clinical evolution of AN patients was assessed for the follow-up of BSFT between Si and Sf using the BMI and scores at the MR scales (five specific and one averaged). The Data for these indices were summarised as median and minimum-maximum intervals. The exact Wilcoxon-Mann-Whitney rank test for paired data was used for each of these indices, and the effect size was computed when a significant evolution was reported. Since several dependent and independent statistical tests were performed simultaneously, we controlled the possible spurious positives using the Bonferroni correction which is the simplest and most conservative approach. Thus for an initial level 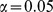 we considered the difference as statistically significant for a corrected threshold 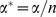 with 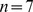 i.e. 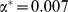.

The evolution of family conflictual interactions during the follow-up of BSFT was tested using the fraction of conflictual communication 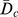 and an exact Wilcoxon-Mann-Whitney rank test for paired data. The relationship between changes in clinical indices (BMI and average score at the MR scales) and changes in conflictual interaction was tested using Pearson's correlation test. For each index 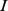, changes were defined as the normalized difference between the score obtained for session Si and session Sf, i.e. 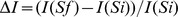.

The evolution of affiliative behaviour between Si and Sf was tested using non-parametric cluster analysis [43]–[45] for conflictual situations using matrices 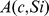 and 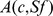 and for non-conflictual situations using matrices 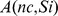 and 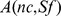. Sources of eye contact (represented as lines in matrices 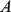) were used to define each cluster so that, for each communicational situation, we tested three clusters, i.e. one for each family member.

The main steps of non-parametric cluster test are illustrated for conflictual situations as an example. For each family, the matrix: 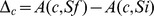 is computed and considered as a paired indicator of changes between Si and Sf. The average matrix 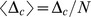 (where 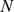 is the number of included families, 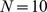) is used as the statistic for further steps. Data are then resampled, keeping the paired characteristics to generate the distribution of the elements of 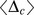 under the null hypothesis, i.e., the absence of a difference between Si and Sf. The experimental value of the elements of 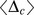 is considered significant if it departs from the permutation distribution for a threshold of 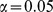.

For each cluster (i.e. family member), a cluster-based statistics was computed as a statistical weight defined as the sum of the differences between the threshold statistics and the experimental statistics if a significant difference has been observed and zero if no significant difference has been observed. A second permutation test is then issued on the cluster-based statistics (i.e. weight). This test allows to determine the distributions under the null hypothesis of the weight of the clusters. Only the clusters for which the cluster-based statistic is higher than that of the permutation distribution (with threshold 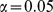) are selected as significant. The same procedure was followed for the non-conflictual situation. This procedure allows to identify specific changes in the eyes contact and to control the family-wise error rate in multiple comparisons.

## Results

### Patients' Evolution between Initial and Final Sessions

Four patients depicted a partly good outcome and six an intermediate outcome. The overall evolution was evaluated using the individual measurements of patients' clinical state (BMI and MR scales). Table 2 shows the changes in individual measures during BSFT for AN patients.

**Table 2:** Changes in individual clinical measures during BSFT sessions for AN patients.

	Initial session			Final session			Wilcoxon test		
	Min.	Med.	Max.	Min.	Med.	Max.	W	p-value	Effect ( 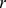 )
BMI	12.50	13.75	15.00	15.00	17.00	18.00	0.000	0.002	0.645
MRnut	0.00	0.00	5.33	4.00	8.16	9.00	0.000	0.002	0.645
MRmens	0.00	0.00	0.00	0.00	0.00	8.00	0.000	0.125	–
MRment	4.00	4.00	4.00	4.00	8.00	8.00	0.000	0.031	–
MRpsex	0.80	1.60	6.80	3.20	6.80	12.00	0.000	0.002	0.645
MRpsoc	0.00	1.60	4.80	5.60	7.20	11.20	0.000	0.002	0.645
MRave	1.12	1.54	3.46	3.52	5.94	9.41	0.000	0.002	0.645

BMI: Body Mass Index; MR: Morgan-Russell Scales; MRnut: nutritional status, MRmens: menstrual functioning, MRment: mental state, MRpsex: psycho-sexual adjustment, MRpsoc: psychosocial adjustment MRave: average of these five previous scores. The p-values should be compared to the Bonferroni corrected threshold 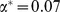 for statistical significance.

At the end of BSFT, the increase in average weight (BMI) was statistically significant. We also observed significant improvement in nutritional (MRnut), psychosexual (MRpsex) and psychosocial (MRpsoc) functioning. No significant difference was observed in mental state (MRment) and menstrual functioning (MRmens). The average MR score (MRave) was significantly improved between Si and session Sf.

### Changes in Family Conflictual Interactions

**Figure 1:**
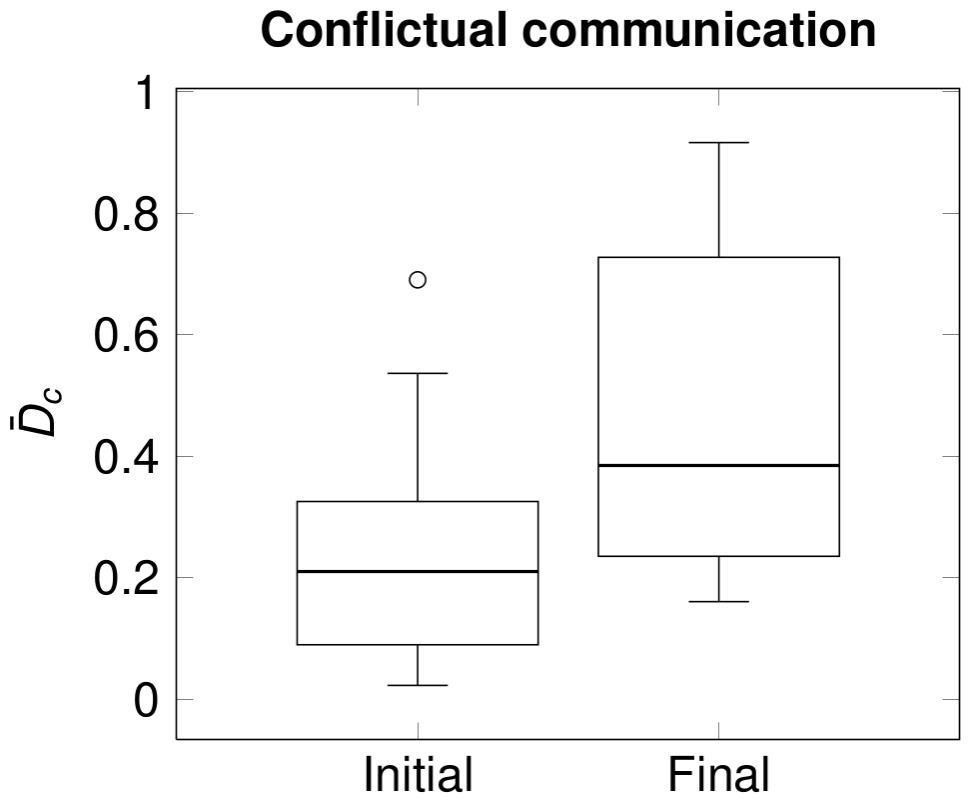
Normalized fraction of duration for the conflictual communications during initial and final sessions of the BSFT. 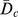
: normalized fraction of duration for the conflictual communications.

At the end of BSFT, there was a significant increase in conflictual communication between family members (Initial session: median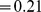, min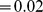 and max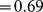; Final session: median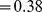, min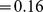 and max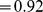; Wilcoxon signed rank test for paired data: 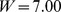, 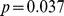, 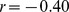; see Figure 1).

**Figure 2:**
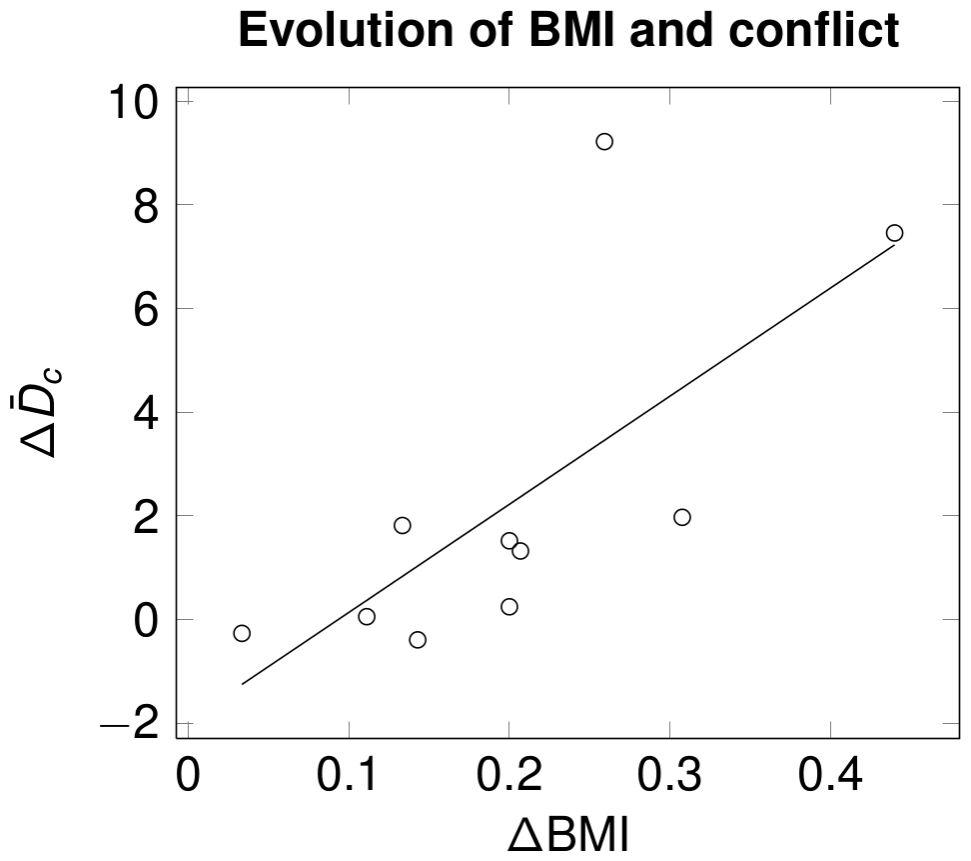
Correlation between the changes in BMI and conflictual communication. Change in BMI is 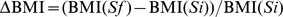. Change in conflictual communication is 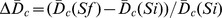. Experimental data (circles) and the regression line are depicted on the figure.

Significant correlation was observed between the normalized change in the percentage of conflictual communication and the improvement in BMI (see Figure 2, 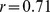, 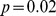). No significant correlation was observed between the normalized change in the percentage of conflictual communication and the average score on the MR scales (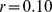, 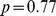).

### Changes in Family Interaction Pattern

The evolution of affiliative nonverbal behaviour between Si and Sf for each family member was tested independently for conflictual and non-conflictual communication using a non-parametric cluster test.

For non-conflictual communication, no statistical weight computed for each cluster exceeded the threshold computed from bootstrap (i.e., patient cluster 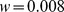, father cluster 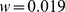, mother cluster 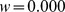 and threshold 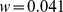). Thus, we could not show any difference in affiliative nonverbal behaviour in the non-conflictual situation.

**Figure 3:**
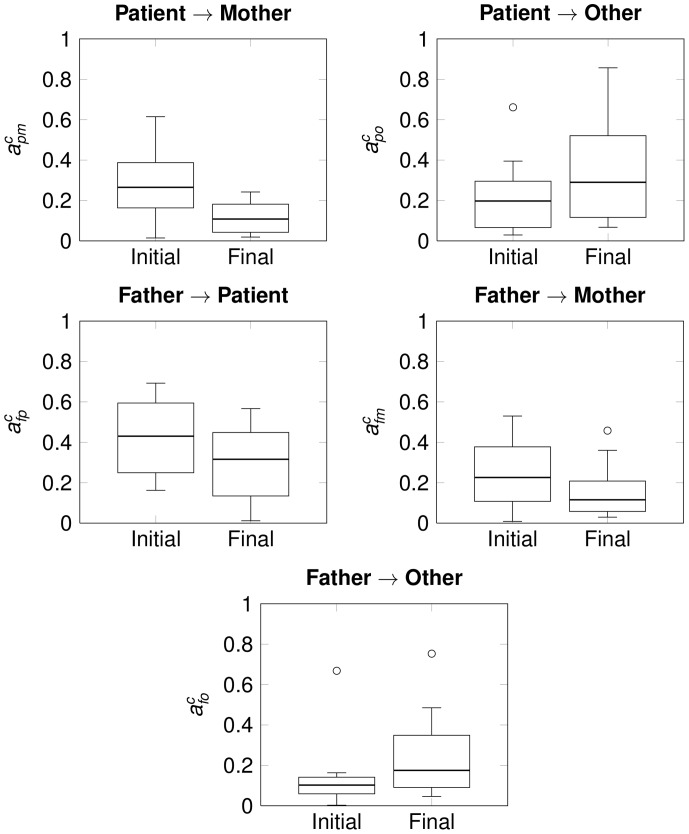
Significant changes between initial and final session of BSFT in affiliative nonverbal behaviour for AN patients and fathers in conflictual situations. Affiliative nonverbal behaviour is measured by eyes contact: 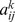 as defined in equation (5).

For conflictual communication, statistical weight for the patient and father (resp. 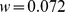 and 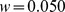) exceeded the permutation threshold (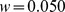) but not for the mother (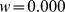). Thus, only the patient and father significantly modified their affiliative nonverbal behaviour between Si and Sf (see Figure 3). For the cluster associated with the father's eye contact, a significant decrease in intra-family affilative behaviour was observed toward the mother and patient as well as an increase in non-affilative behaviour. For the cluster associated with the patient's eye contact, a decrease in intra-family affiliative behaviour towards the mother was observed as well as an increase in non-affiliative behaviour.

## Discussion

### Conflictual Communication

The present study assessed the evolution of clinical symptoms of AN patients and conflictual communication in their families during BSFT. The observation of changes in clinical indices between the first and the last session of BSFT in AN patients demonstrates an improvement in clinical symptoms and in psychosocial functioning of restrictive-type eating disorders as measured by BMI and the MR scales. In addition to these clinical improvements, a significant increase in conflictual communication in the families of AN patients was observed. This increase in conflictual communication was significantly correlated with the improvement of BMI.

Families of AN patients have been characterised by deficit of emotional expressiveness, especially concerning conflicts and a lack of conflict resolution [37], [46]–[48]. Our observation that an increase in conflictual communication correlated with BMI improvement suggests that conflict management is a clinically meaningful index of family functioning in the case of AN. It is in agreement with the previous results in the literature and are together consistent with the importance of the specific monitoring of conflict management in BSFT. Nevertheless, our result showed that increase in conflictual communication is not significantly correlated with the changes in the average score on the MR scales, suggesting that family therapy between 5 and 7 sessions is a too short period in outpatient treatment. In inpatient treatment, it has been shown that increase in conflictual communication predicted the improvement in the nutritional state of AN patients at a middle phase of therapy family (sessions 5 to 8) and the improvement in the psychological states at the end of family therapy (sessions 9 to 14) [16].

Three studies provide the most direct comparison with the present one. First, Robin et al. [20] showed a reduction in conflict related to eating in problem solving tasks at a 1-year follow-up of family therapy, then Eisler et al. [5] supported that the decrease in hostile parental attitudes related to the illness of patient are improvement factors of eating symptoms, and finally, Shugar et al. [16] reported an increase in overt communication of confrontation at a 1-year follow-up of family therapy. These studies have used self-report scales [16], [20] and observational methods of dyadic interactions [5]. Because, self-report scales measure the family members' representation of family interaction and may not correspond to an observer's report, in our study, we used data based on direct measurements of family interactions. Furthermore, in observational methods of interaction, the experimenter assesses the state of mind and attitudes of a particular family member in the absence of the other family members, but the emotional involvement of each member is determined by the interactions between all members of the family [49], [50]. In the present study, however, the measurement of triadic interactions between the patient, the mother and the father is the center of the analysed data. According to family systems theory, the observational measures of family interaction are important indicators for determining therapeutic interventions in the family therapy [38], [50] and our study reinforce this statement.

### Affiliative Behaviour

**Figure 4:**
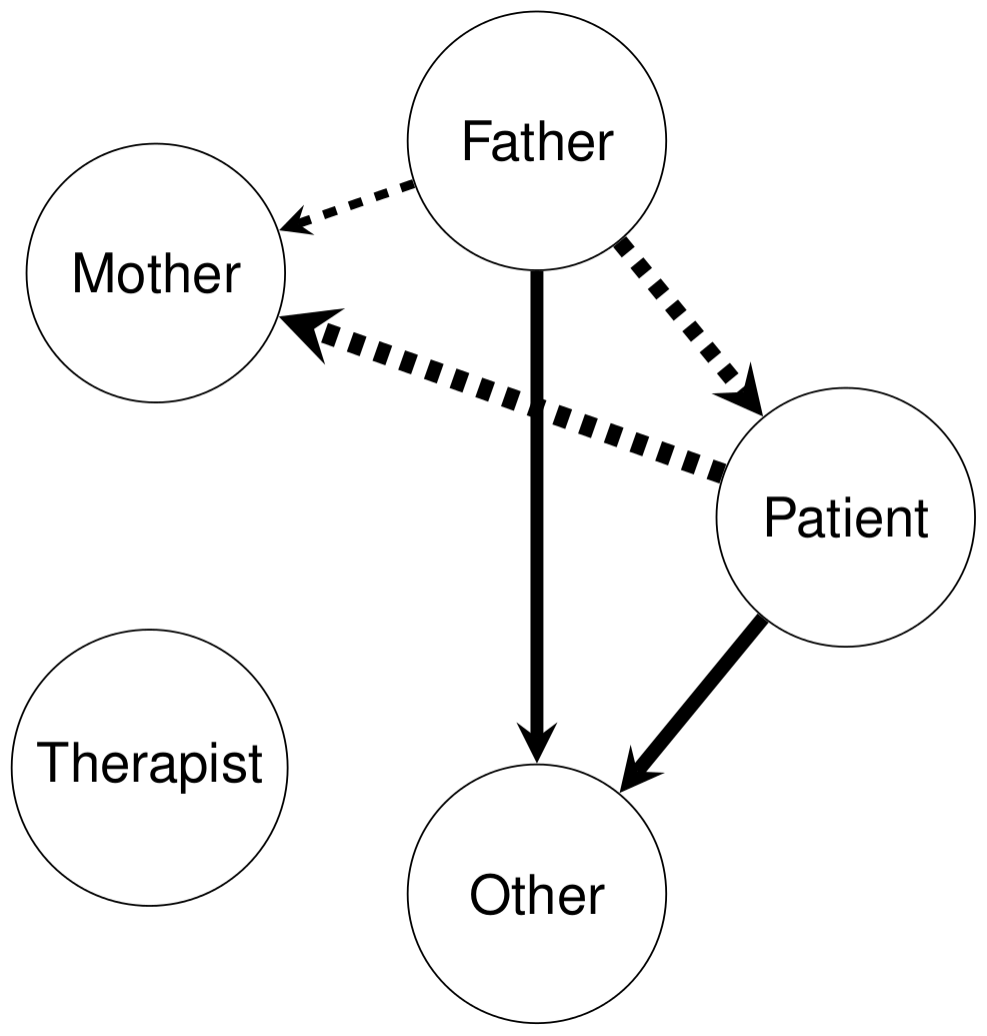
Graphical representation of changes in affiliative behaviour in conflictual communication during family therapy between initial and final session. Dashed line: decrease of behaviour; continuous line: increase of behaviour. Thickness of the arcs are proportional to the value of the changes.

The second central goal of the present study was inspired by clinical research which have shown that affiliative behaviour is a sign of emotional engagement between participants [9], [27]. We thus used the affiliative behaviour measured by eye contact to reveal changes in emotional involvement during conflictual communication in family of AN patients during BSFT. The findings from this study showed, in conflictual situations only, a decrease in intra-family affiliation expressed by the father and the patient after a BSFT follow-up. These changes are paralleled with an increase in non-affiliative behaviour for these two family members (see figure 4). In contrast, no significant difference is observed in affiliative nonverbal behaviour in mother. These results demonstrate that the impact of the BSFT differs between members of a family: the AN patient and the father have established a new form of emotional functioning with a decrease in emotional involvement, whereas the mother does not change her affiliative behaviour and, thus, her emotional involvement. The persistence of nonverbal emotional involvement expressed by the mother during conflicting communication may be determined by a high level of anxiety and emotional distress [51], [52] despite improved clinical symptoms measured by the BMI. The nonverbal distancing behaviour in patients and fathers may reflect adaptive modes of coping with interpersonal stress. Moreover, it has been shown that open-minded communication and conflict resolution in the father-daughter relationship constitute a protective factor against AN [53].

These results can be enlightened by recent studies which show that the ability to inhibit the gaze predicts adaptive social functioning in adulthood [30]. A greater ability to represent the mental states of others and a high level of self-other differentiation within a close relationship context are also related to gaze regulation [28]. These results and ours demonstrate that affiliative nonverbal behaviour is an important variable that need to be taken into account in family therapy.

### Conclusion

Although the majority of studies of family therapy focus on verbal behaviour [6], [16], [20], the present results establish that the affiliative nonverbal behaviour associated with verbal interactions is an important element for understanding family functioning. Thus, the study of the combination between verbal and nonverbal communication in family therapy can represent an important step in the understanding of the mechanisms of therapeutic change. However, our sample is small and limit the generalizability of our results. Moreover, our sample concerns young adults and our findings may not be generalizable to younger AN adolescents. Indeed, the comparison between younger AN adolescents and young AN adults could be important for understanding the family interactions and for determining therapeutic interventions in the family therapy according to age and the duration of the AN [54].

Several perspectives can be envisioned. First, a further study after the end of the family treatment is needed to verify the stability of the new pattern of communication and affiliative behaviour in conflictual situations and the generalisation of these new abilities to other peer interactions. Moreover, the specificity of changes in affiliative nonverbal behaviour should be evaluated in regard to other highly emotional situations. Finally, the behavior of the therapist is another putative element of the therapeutic changes which cannot be studied in the present article. The same quantification of verbal and nonverbal behavior should be applied to the therapist and be included in the study of the global pattern of interactions to infer his/her implication in the therapeutic changes.
